# GTDB: an integrated resource for glycosyltransferase sequences and annotations

**DOI:** 10.1093/database/baaa047

**Published:** 2020-06-16

**Authors:** Chenfen Zhou, Qingwei Xu, Sheng He, Wei Ye, Ruifang Cao, Pengyu Wang, Yunchao Ling, Xing Yan, Qingzhong Wang, Guoqing Zhang

**Affiliations:** 1 National Genomics Data Center, Bio-Med Big Data Center, Key Laboratory of Computational Biology, CAS-MPG Partner Institute for Computational Biology, Shanghai Institute of Nutrition and Health, University of Chinese Academy of Sciences, Chinese Academy of Sciences, 320 Yueyang Road, Xuhui, Shanghai 200031, China; 2 College of Computer, Hubei University of Education, 129 Second Gaoxin Road, Wuhan Hi-Tech Zone, Wu Han 430205, China; 3 School of Life Science and Technology, ShanghaiTech University, 393 Middle Huaxia Road, Pudong, Shanghai 201210, China; 4 CAS-Key Laboratory of Synthetic Biology, CAS Center for Excellence in Molecular Plant Sciences, Institute of Plant Physiology and Ecology, Chinese Academy of Sciences, 300 Fenglin Road, Xuhui, Shanghai 200032, China

## Abstract

Glycosyltransferases (GTs), a large class of carbohydrate-active enzymes, adds glycosyl moieties to various substrates to generate multiple bioactive compounds, including natural products with pharmaceutical or agrochemical values. Here, we first collected comprehensive information on GTs, including amino acid sequences, coding region sequences, available tertiary structures, protein classification families, catalytic reactions and metabolic pathways. Then, we developed sequence search and molecular docking processes for GTs, resulting in a GTs database (GTDB). In the present study, 520 179 GTs from approximately 21 647 species that involved in 394 kinds of different reactions were deposited in GTDB. GTDB has the following useful features: (i) text search is provided for retrieving the complete details of a query by combining multiple identifiers and data sources; (ii) a convenient browser allows users to browse data by different classifications and download data in batches; (iii) BLAST is offered for searching against pre-defined sequences, which can facilitate the annotation of the biological functions of query GTs; and lastly, (iv) GTdock using AutoDock Vina performs docking simulations of several GTs with the same single acceptor and displays the results based on 3Dmol.js allowing easy view of models.

## Introduction

**Figure 1:**
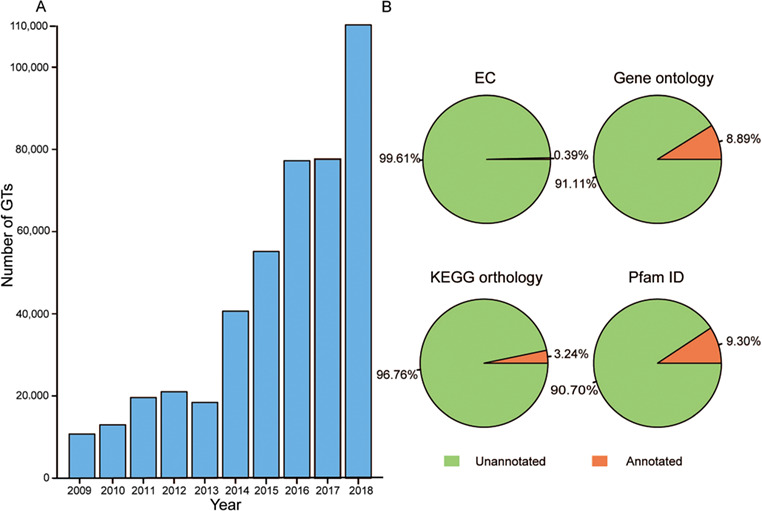
Growth of GT number over the past decade and the proportion of annotation data before predictions. (**A**) Number of GTs has increased rapidly in the past 10 years. (**B**) Orange represents GTs with the corresponding annotated data and green is GTs without the corresponding annotated data.

Glycosyltransferases (GTs) are an important group of enzymes that can catalyze the transfer of activated sugar residues onto a wide range of carbohydrates or non-carbohydrate acceptors, generating a remarkable amount of structural diversity in biological systems (1). These enzymes commonly exist in different species from prokaryotes to eukaryotes. For prokaryotes, heptosyltransferases I–IV sequentially add sugar moieties to generate the core of lipopolysaccharides in the cell-surface components of Gram-negative bacteria (2). In *Arabidopsis thaliana*, AtGLCAT14A can modify beta-1,6-linked galactan and beta-1,3-linked galactan present in type II arabinogalactan (3); AtGAUT1 can catalyze the transfer of galacturonic acid from uridine 5′-diphosphogalacturonic acid onto the polysaccharide homogalacturonan in pectin biosynthesis (4); UGT78D1, UGT78D2 and UGT78D3 can catalyze the first 3-O-glycosylation in the biosynthesis of the flavonoids kaempferol, quercetin and isorhamnetin (5). In humans, GnT-V can catalyze the formation of β1,6-GlcNAc branching, GnT-III can transfer the GlcNAc to the β-mannose residue of N-glycans and Fut8 can transfer a fucose moiety from GDP-β-L-fucose to the innermost GlcNAc residue in an N-glycan, which all play key roles in cancer progression and treatment (6). Overall, GTs are involved in a variety of critical biological activities, including cell wall construction, natural product formation, cancer metastasis and suppression (7). At present, the number of GTs has increased rapidly over the past 10 years because of high-throughput transcriptomic sequencing technologies (Figure 1A), but functional information about these novel proteins remains to be further annotated (Figure 1B). It appears that the gap between protein sequences and identified functions often leads to much trouble for researchers studying GTs (8–9).

To date, several comprehensive resources regarding GTs have been constructed and have facilitated the application of GTs to a variety of problems. Carbohydrate-active enzymes (CAZy) (http://www.cazy.org) depicts families of enzymes that degrade, modify or create glycosidic bonds, including GTs, glycoside hydrolases, polysaccharide lyases, carbohydrate esterases, auxiliary activities and carbohydrate-binding modules (9). The CAZy database focuses on the classification of GTs based on their sequence similarity and displays basic information about entries, including the protein names, organism, enzyme commission (EC) numbers and external database accessions. However, CAZy does not have a centralized sequence search function nor does it provide direct download access for CAZy sequences or annotations, limiting its utility. KEGG GLYCAN (https://www.kegg.jp/kegg/glycan/) (10), a resource for carbohydrate structures where entries are organized by functional ortholog groups rather than single specific GT, covers a subset of GTs classified by their synthesis of glycosyl bonds. Moreover, PlantCyc (https://www.plantcyc.org) (11), dbCAN-seq (http://bcb.unl.edu/dbCAN_seq/index.php) (12), the Rice GT Database (http://ricephylogenomics.ucdavis.edu/cellwalls/gt/index.shtml) (13) and CSDB_GT (http://csdb.glycoscience.ru/gt.html) (14–15) concentrate on several specific species yet cannot be applied to all living domains. Therefore, an integrated GT resource for multiple species is needed and could help researchers access sequences and annotation data in a centralized location rather than using various scattered databases.

Considering the above points, we therefore constructed the GTs database (GTDB), which has combined a variety of contents from well-known public databases including CAZy, UniProt (16), KEGG (17) and MetaCyc (18), as well as pre-computed annotations derived using DIAMOND (double index alignment of next-generation sequencing data) (19), eggNOG-mapper (v2.0.0) (20), HMMER (v3.2.1) (21), interactive tools with BLAST (v2.7.1) (22) and docking modeling using AutoDock Vina (v1.1.2) (23). GTDB displays detailed information on each GT from multiple aspects, including sequences, structures, protein family classifications, protein functions, enzyme reactions and external links. If a protein annotation is predicted *in silico*, these results are labeled with corresponding bioinformatics methods. Thus, GTDB provides search methods via different data sources (third-party database, predictions in GTDB) to meet users’ distinct demands. It also supplies batch download service for annotated data grouped by several characteristic sets. In addition, GTDB is free and available at https://www.biosino.org/gtdb/.

## Materials and Methods

### Data sources

In order to broad our understanding of GTs, GTDB merged diverse and valuable data from several well-defined databases listed in Table 1. Basic information on GTs, including their protein names, organism, protein Genbank accessions, UniProt accessions, EC numbers, PDB codes, GT families and mechanisms, was obtained from the CAZy (http://www.cazy.org/) database, and protein names were kept in the original style in this database that uses a combination of trivial names, gene identifications and locus tags. Gene information on GTs, including gene symbol and gene ID, was integrated using data from NCBI Gene (https://www.ncbi.nlm.nih.gov/gene/) (24). The protein sequences and related CDS were from NCBI Protein (https://www.ncbi.nlm.nih.gov/protein/) and Nucleotide (https://www.ncbi.nlm.nih.gov/nuccore), respectively. The species information, namely taxonomy ID, came from NCBI Taxonomy (https://www.ncbi.nlm.nih.gov/taxonomy/) (25). The information on references, e.g. author lists, article titles and PMID, was gleaned from NCBI PubMed (https://www.ncbi.nlm.nih.gov/pubmed/) (26). We then extracted protein characteristics of GTs, which included tissue specificity, subcellular locations and kinetics information from UniProt knowledgebase (https://www.uniprot.org/). The 3D structure information, including experimental methods used, resolution, ligand and specific references, was acquired from the RCSB Protein Data Bank (http://www.rcsb.org/) (27). Pathway, enzyme kinetic parameters (KM, KCAT, VMAX), optimum conditions (PH, temperature) and catalytic reactions were from MetaCyc (https://metacyc.org/). All protein sequences from MetaCyc act as a reference dataset when we applied DIAMOND. EC numbers of the GTs in GTDB were mostly from BRENDA (https://www.brenda-enzymes.org) (28), which is a comprehensive enzyme information system. Information on protein domains was extracted from the Pfam database (http://pfam.xfam.org) (29), which is a large collection information grouped by protein family. Also, the Pfam A set functioned as a target database for the use of HMMER3 (Figure S1).

**Table 1:** List of databases and algorithms used in GTDB

Database or algorithm	Contents for GTDB	Version
CAZy	Genbank Accession, Uniprot Accession, PDB ID, GT name, EC number, Mechanism, GT Family, Organism, Classification,3D Structure Status	17 April 2019
NCBI Protein	Protein sequences	April 2019
NCBI Nucleotide	Coding region sequences	April 2019
NCBI Gene	Gene symbol, Gene ID	April 2019
NCBI PubMed	Reference	April 2019
NCBI Taxonomy	Taxonomy ID	April 2019
RCSB PDB	Literature, Ligand ID, Ligand name, Resolution, Method	April 2019
UniProt	Gene ontology, KEGG orthology, Reaction, Tissue specificity, Developmental stage, Subcellular location, Protein sequences, Kinetics, PH-opt, Temperature-opt, Enzyme ID, Pfam ID	April 2019
Metacyc	Reaction, Reaction ID, VMAX, KCAT, KM, PH-opt, Temperature-opt	23.2
KEGG	Diseases Involved	1 August 2018
EggNOG	Gene ontology, KEGG orthology	5.0.0
DIAMOND	EC number, pathway ID (Metacyc), enzyme ID (Metacyc)	
HMMER	Pfam ID, Pfam name	3.2.1

### Function annotations

By means of the precomputed eggNOG database clusters and phylogenies analysis, eggNOG-mapper can annotate large sets of proteins via fast orthology assignments (20). Here, 554 892 GT sequences were annotated using eggNOG-mapper (v2.0.0) and the eggNOG database (v5.0.0) (30). Due to the large sequence sets and limited time schedule, DIAMOND mode was selected, using 0.001 e-value threshold, two threads and three alignments reported.

The high-throughput program DIAMOND can align DNA reads or protein sequences against a protein reference database with high speed and high sensitivity (19). It was implemented to annotated enzymology information and metabolic pathways of GTs in GTDB with an e-value cutoff of 0.001 and a maximum number of target sequences reported of 1. For EC number annotation, all protein sequences in the UniProt Swiss-Prot database (version April 2019) were downloaded as a reference database. For pathway annotation, all protein sequences in MetaCyc (v23.2) were used as references. The pathway contents, enzyme kinetic parameters and catalytic reactions of GTs were all integrated into GTDB based on the enzyme IDs from MetaCyc. Additionally, HMMER3, using Pfam A data and e-value threshold of $1\times{10}^{-5}$, was used to detect the signature domains of GTs (Figure S1).

### Tools developed

We developed BLAST and GTdock tools based on known GTs in GTDB. BLAST was carried out via NCBI-BLAST 2.7.1, with default expect threshold of 10 and a maximum of 250 aligned sequences (31). The entire set of protein sequences in GTDB was used as a target database. Other optional reference databases are also available, including datasets grouped by organism or obtained from different identification means (UniProt Swiss-Prot or TrEMBL). To start a BLAST search, users can paste or upload no more than 10 query sequences at a time.

To predict the possible molecular interactions between GTs and a given glycosyl-acceptor, we developed GTdock tools. To use this function, users should first acquire structure files of macromolecular proteins and small molecular acceptors. For small molecular acceptors, users can select from any of 44 confirmed glycosyl-acceptors provided by UniProt or upload a custom acceptor in SDF format. For macromolecular proteins, the maximum number of structural files allowed is 10. Candidate proteins for docking should only be those with identities >40% and an expected value less than 0.01 in a BLAST comparison with all GTs in GTDB. Finally, Autodock Vina performs molecular docking, where the parameter of each protein center was calculated using the central position of the corresponding molecule and the box dimensions in x, y, z were all set to 40. Once the above analysis was finished, the docking results for each GT with highest interactive score are automatically e-mailed to users.

### Website design and database backend

GTDB merges diverse and heterogeneous datasets coming from distinct communities and deposits them in MongoDB after Extract-Transform-Load processes. Compared with traditional relational database management systems, MongoDB provides more flexible ways for necessary future expansion. GTDB was developed using java SpringBoot and integrated a couple of enhanced utilities for providing visual presentations of GTs data. For instance, 3D structures of GTs are represented in 3Dmol.js (http://3dmol.csb.pitt.edu/) (32), and a set of statistical charts were developed based on ECharts (http://echarts.baidu.com/index.html). In addition, we encapsulated Blast and GTdock services as independent docker applications (https://www.docker.com/), which enabled easier deployment and quick updates.

## Results

### Overview and data summary

**Figure 2:**
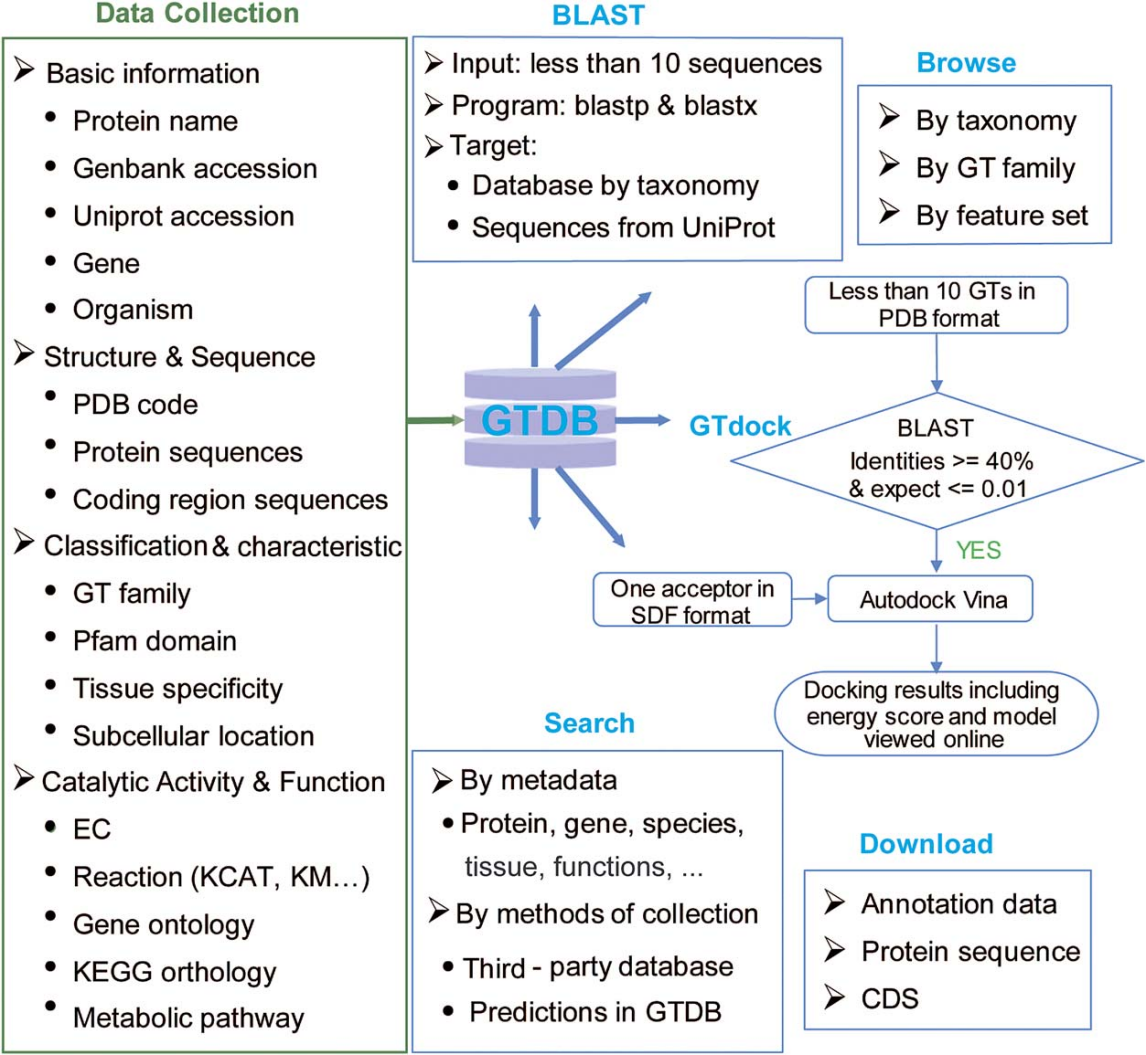
Content and services in GTDB. The left panel is the database content, which includes a variety of information on GTs from well-known databases and predictions. The right panel is the web service provided by GTDB, including Search, Browse and Download models that can be used to access the database flexibly, as well as two useful tools for searching similar GTs by sequence (BLAST) or performing GT docking (GTdock).

GTDB was built to offer an integrated resource that incorporates GT sequences and annotations across multiple species, and a user-friendly web interface that allows data retrieval using different types of identifiers, searching against pre-organized protein sequences by BLAST and performing molecular docking of one acceptor with some GTs via GTdock (Figure 2).

GTDB collects a large number of sets of GT data that in total consist of 520 179 GTs with 554 892 protein sequences, approximately 21 647 species, 33 720 genes, 394 full EC numbers, 4788 gene ontology (GO) terms and 10 271 references. It covers 105 GT families according to the CAZy database (version 17 April 2019), where GT0 indicates non-classified sequences and GT2 harbors the most abundant number of GTs (159521). In terms of annotated content, 8.9% of GTs have GO terms from third-party database and 35.3% GTs’ GO terms were further predicted in GTDB. ‘Transferase activity (GO:0016740),’ which is one of the main characteristics of GTs, accounts for 44.1% and 89.5% in the two previously mentioned sources, respectively. For KEGG orthology (KO) annotations, 3.2% of GTs have KO from knowledge-based resources, and 68.3% GTs’ KO were widely annotated in GTDB. Notably, there are five KOs—k02563, k05366, k00688, k07011 and k03814—that are all associated strongly with carbohydrate transport and metabolism, making up a large proportion in the total number of KOs from different methods. For domain prediction, 9.3% of GTs’ Pfam data were extracted from UniProt and 79.6% of these domains were extended predictions. All relevant data can be viewed on the ‘Statistics’ page of GTDB.

### Search

**Figure 3:**
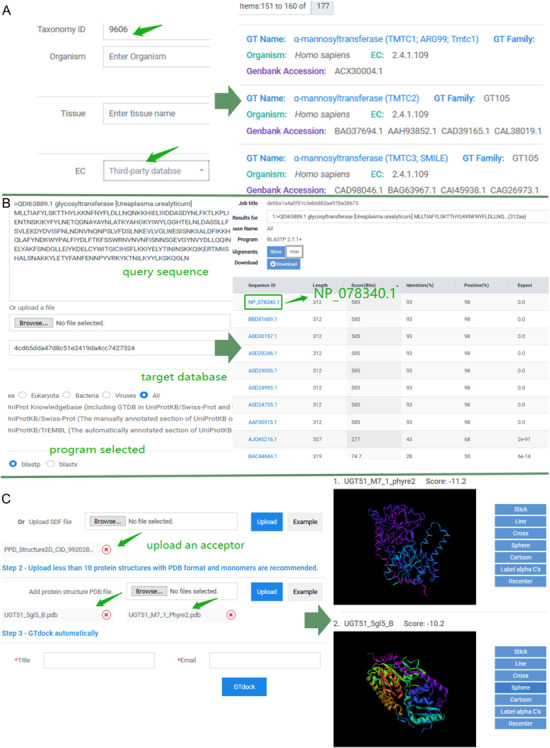
Examples of application of GTDB. (**A**) An example of advanced search. Input ‘9606’ into the ‘Taxonomy ID’ box of taxonomy module and choose ‘Third-party database’ for EC in the function module. Subsequently, there were totally of 177 entries in GTDB. Users can select any of them to view the details. (**B**) An example of sequence search. Paste the sequence of ‘QDI63889.1’ and select the ‘All’ as the target database. The default 250 aligned sequences will be displayed on the BLAST results page. (**C**) An example of the use of GTdock. Enter the example data on the GTdock page and the result link is mailed to the user. Note that the docking algorithm is non-deterministic of Vina, so the minimum score of enzyme-acceptor pair will produce some nuances in different operations (23).

Keyword search and sequence search were both implemented in GTDB, where keyword search consists of three accesses. First, users can input a GT name, protein Genbank accession, or UniProt accession on the top query bar using pre-defined search criteria. Second, users can click on any GT name directly in a word cloud on ‘Home’ page to start a quick investigation of interested GTs. Lastly, an advanced search on the ‘Search’ page allows more sophisticated searching, where various query items are pre-classified into several categories, including protein information (GT name, Genbank accession, UniProt accession or PDB ID), gene information (gene ID and gene symbol), taxonomy (taxonomy ID or organism), tissue, functions (EC, GO, KO, Pfam accession or pathway) and reaction information (reaction, KCAT, KM, VMAX, PH-opt and temperature-opt). Queries of ‘function’ and ‘reaction’ provide the label of the data source (third-party database or predictions in GTDB), which all assist the users to address specific search concerns accurately. An example of advanced search result is shown in Figure 3A.

Sequence searches using BLAST can be helpful for analyzing new or similar GTs in GTDB. For instance, GT (*Ureaplasma urealyticum*) (Genbank accession: QDI63889.1) is new for GTDB because it was newly created on 9 July 2019 in the NCBI Protein resource. While submitting QDI63889.1 as a query sequence and choosing ‘All’ as the target database, a sorted table of results with links of each aligned entry is generated. As the best alignment results from this search (NP_078340.1 identities = 93% expect = 0) (33) indicates GT (*U. urealyticum*) may belong to GT2 family and has a catalytic mechanism of inverting. The BLAST results are shown in Figure 3B.

### Implementation of GTdock

Considering that GT structure can provide suitable catalytic sites and the special microenvironment for glycosylation reactions, we included binding mode prediction software in GTDB. AutoDock Vina has the advantages of both high speed and high accuracy in molecular docking, so we integrated Autodock Vina and BLAST to build the GTdock pipeline. GTdock can fulfill molecular docking with an acceptor and several GTs at one time and display those results based on 3Dmol.js that provides an interactive interface to view the binding modes.

Here, we used UDT51 as an example to illustrate the function of GTdock. In *Saccharomyces cerevisiae S288c*, UGT51 can catalyze its natural substrate—ergosterol—with very high activity. Researchers have found that UGT51 can also catalyze protopanaxadiol (PPD) with ~13% of the conversion ratio as compared with ergosterol. They then reported mutant M7_1 (S801A/L802A/V804A/K812A/E816K/S849A/N892D), which had an 1800-fold activity improvement for the unnatural substrate PPD (34). A 3D structure of M7_1 was then obtained from Phyre2 with normal mode (35). The 3D structure of PPD (PubChem CID: 9920281) in SDF format and UGT51 (PDB code: 5GL5_B), M7_1 in PDB format were all submitted to GTdock. At last, each binding mode with its lowest energy score is displayed on the result page (Figure 3C). In Autodock Vina, the more negative the score, the better the docking results. In this case, the molecular docking results indicated that a uniquely changed interaction network in this enzyme may have an effect on its substrate preference (36). Although GTdock can provide some help for studying the binding mode between GTs and acceptors, users should also pay attention to distinguish the relative correct conformation according to their own research purposes and knowledge for further analysis (More examples of GTdock running are shown in Figure S2).

## Discussion

In summary, GTDB is an integrated repository with various information on GTs from multiple species. It includes information such as protein sequences, tertiary structures, catalytic activities and function annotations. GTDB not only harbors the common contents from third-party databases but also includes predictions *in silico*. Moreover, BLAST and GTdock as useful tools are incorporated into GTDB that can be used to explore the related characteristics of GTs from sequences and structures, respectively. GTDB is an easy-to-use website and is convenient for users wishing to search and download pre-defined datasets from the database. Nevertheless, GTDB has some shortcomings, for example, GTdock needs PDB files from extra protein structure modeling websites if there are no existing 3D structures, and bulk operations on more than 10 sequence and structure files are also required.

In the future, we will regularly update the GTDB datasets based on the latest versions of other well-known databases described in the ‘Data source’ section. Meanwhile, we will consider integrating other crucial data, including additional organism contents, transcriptome data and protein sequence characteristics of GTs. For catalytic reactions of GTs, we would like to sort and correct predicted catalytic reactions (239 417, 46%) manually based on publications. We will also improve the GTdock tool to achieve more accurate docking results by integrating an improved center finding algorithm. To conclude, GTDB will facilitate further identification of GTs and understanding of the vital roles that GTs play in glycobiology, synthetic biology, drug design and development.

## Supplementary data

Supplementary data are available at *Database* online.

## Author contributions

C.Z. performed the analyses, data collection and most web design and drafted the paper. Q.X. set up the database and participated in web design and S.H. and W.Y. constructed the GTdock tool pipeline and participated the website design. R.C. contributed to data collection. P.W. took part in the database building. Y.L., X.Y. and Q.W. edited the final manuscript. G.Z. conceived the study and edited the final manuscript. All authors read and approved the final manuscript.

## Supplementary Material

supplymentary_file_baaa047

## References

[1] Lairson L.L., Henrissat B., Davies G.J. et al. (2008) Glycosyltransferases: structures, functions, and mechanisms. Annu. Rev. Biochem., 77, 521–555. 18518825 10.1146/annurev.biochem.76.061005.092322

[2] Cote J.M. and Taylor E.A. (2017) The glycosyltransferases of LPS core: a review of four heptosyltransferase enzymes in context. Int. J. Mol. Sci., 18, E2256. 29077008 10.3390/ijms18112256 PMC5713226

[3] Knoch E., Dilokpimol A., Tryfona T. et al. (2013) A beta-glucuronosyltransferase from *Arabidopsis thaliana* involved in biosynthesis of type II arabinogalactan has a role in cell elongation during seedling growth. Plant J., 76, 1016–1029. 24128328 10.1111/tpj.12353

[4] Sterling J.D., Atmodjo M.A., Inwood S.E. et al. (2006) Functional identification of an *Arabidopsis pectin* biosynthetic homogalacturonan galacturonosyltransferase. Proc. Natl. Acad. Sci. U. S. A., 103, 5236–5241. 16540543 10.1073/pnas.0600120103 PMC1458824

[5] Yonekura-Sakakibara K., Tohge T., Matsuda F. et al. (2008) Comprehensive flavonol profiling and transcriptome coexpression analysis leading to decoding gene-metabolite correlations in *Arabidopsis*. Plant Cell, 20, 2160–2176. 18757557 10.1105/tpc.108.058040 PMC2553606

[6] Taniguchi N. and Kizuka Y. (2015) Glycans and cancer: role of N-glycans in cancer biomarker, progression and metastasis, and therapeutics. Adv. Cancer Res., 126, 11–51. 25727145 10.1016/bs.acr.2014.11.001

[7] Liang D.M., Liu J.H., Wu H. et al. (2015) Glycosyltransferases: mechanisms and applications in natural product development. Chem. Soc. Rev., 44, 8350–8374. 26330279 10.1039/c5cs00600g

[8] Cantarel B.L., Coutinho P.M., Rancurel C. et al. (2009) The carbohydrate-active enzymes database (CAZy): an expert resource for glycogenomics. Nucleic Acids Res., 37, D233–D238. 18838391 10.1093/nar/gkn663 PMC2686590

[9] Lombard V., Ramulu H.G., Drula E. et al. (2014) The carbohydrate-active enzymes database (CAZy) in 2013. Nucleic Acids Res., 42, D490–D495. 24270786 10.1093/nar/gkt1178 PMC3965031

[10] Hashimoto K., Goto S., Kawano S. et al. (2006) KEGG as a glycome informatics resource. Glycobiology, 16, 63R–70R. 10.1093/glycob/cwj010 16014746

[11] Zhang P., Dreher K., Karthikeyan A. et al. (2010) Creation of a genome-wide metabolic pathway database for Populus trichocarpa using a new approach for reconstruction and curation of metabolic pathways for plants. Plant Physiol., 153, 1479–1491. 20522724 10.1104/pp.110.157396 PMC2923894

[12] Huang L., Zhang H., Wu P. et al. (2018) dbCAN-seq: a database of carbohydrate-active enzyme (CAZyme) sequence and annotation. Nucleic Acids Res., 46, D516–D521. 30053267 10.1093/nar/gkx894 PMC5753378

[13] Cao P.J., Bartley L.E., Jung K.H. et al. (2008) Construction of a rice glycosyltransferase phylogenomic database and identification of rice-diverged glycosyltransferases. Mol. Plant, 1, 858–877. 19825588 10.1093/mp/ssn052

[14] Egorova K.S. and Toukach P.V. (2016) CSDB_GT: a new curated database on glycosyltransferases. Glycobiology, 27, 285–290. 28011601 10.1093/glycob/cww137

[15] Egorova K.S., Knirel Y.A. and Toukach P.V. (2019) Expanding CSDB_GT glycosyltransferase database with *Escherichia coli*. Glycobiology, 29, 285–287. 30759212 10.1093/glycob/cwz006

[16] UniProt Consortium (2019) UniProt: a worldwide hub of protein knowledge. Nucleic Acids Res., 47, D506–D515. 30395287 10.1093/nar/gky1049 PMC6323992

[17] Kanehisa M., Sato Y., Furumichi M. et al. (2019) New approach for understanding genome variations in KEGG. Nucleic Acids Res., 47, D590–D595. 30321428 10.1093/nar/gky962 PMC6324070

[18] Caspi R., Billington R., Fulcher C.A. et al. (2018) The MetaCyc database of metabolic pathways and enzymes. Nucleic Acids Res., 46, D633–D639. 29059334 10.1093/nar/gkx935 PMC5753197

[19] Buchfink B., Xie C. and Huson D.H. (2015) Fast and sensitive protein alignment using DIAMOND. Nat. Methods, 12, 59–60. 25402007 10.1038/nmeth.3176

[20] Huerta-Cepas J., Forslund K., Coelho L.P. et al. (2017) Fast genome-wide functional annotation through orthology assignment by eggNOG-mapper. Mol. Biol. Evol., 34, 2115–2122. 28460117 10.1093/molbev/msx148 PMC5850834

[21] Mistry J., Finn R.D., Eddy S.R. et al. (2013) Challenges in homology search: HMMER3 and convergent evolution of coiled-coil regions. Nucleic Acids Res., 41, e121. 23598997 10.1093/nar/gkt263 PMC3695513

[22] Johnson M., Zaretskaya I., Raytselis Y. et al. (2008) NCBI BLAST: a better web interface. Nucleic Acids Res., 36, W5–W9. 18440982 10.1093/nar/gkn201 PMC2447716

[23] Trott O. and Olson A.J. (2010) AutoDock Vina: improving the speed and accuracy of docking with a new scoring function, efficient optimization, and multithreading. J. Comput. Chem., 31, 455–461. 19499576 10.1002/jcc.21334 PMC3041641

[24] Brown G.R., Hem V., Katz K.S. et al. (2015) Gene: a gene-centered information resource at NCBI. Nucleic Acids Res., 43, D36–D42. 25355515 10.1093/nar/gku1055 PMC4383897

[25] Federhen S. (2012) The NCBI Taxonomy database. Nucleic Acids Res., 40, D136–D143. 22139910 10.1093/nar/gkr1178 PMC3245000

[26] Sayers E.W., Agarwala R., Bolton E.E. et al. (2019) Database resources of the National Center for Biotechnology Information. Nucleic Acids Res., 47, D23–D28. 30395293 10.1093/nar/gky1069 PMC6323993

[27] Burley S.K., Berman H.M., Bhikadiya C. et al. (2019) RCSB Protein Data Bank: biological macromolecular structures enabling research and education in fundamental biology, biomedicine, biotechnology and energy. Nucleic Acids Res., 47, D464–D474. 30357411 10.1093/nar/gky1004 PMC6324064

[28] Jeske L., Placzek S., Schomburg I. et al. (2019) BRENDA in 2019: a European ELIXIR core data resource. Nucleic Acids Res., 47, D542–D549. 30395242 10.1093/nar/gky1048 PMC6323942

[29] El-Gebali S., Mistry J., Bateman A. et al. (2019) The Pfam protein families database in 2019. Nucleic Acids Res., 47, D427–D432. 30357350 10.1093/nar/gky995 PMC6324024

[30] Huerta-Cepas J., Szklarczyk D., Heller D. et al. (2019) eggNOG 5.0: a hierarchical, functionally and phylogenetically annotated orthology resource based on 5090 organisms and 2502 viruses. Nucleic Acids Res., 47, D309–D314. 30418610 10.1093/nar/gky1085 PMC6324079

[31] Mcginnis S. and Madden T.L. (2004) BLAST: at the core of a powerful and diverse set of sequence analysis tools. Nucleic Acids Res., 32, W20–W25. 15215342 10.1093/nar/gkh435 PMC441573

[32] Rego N. and Koes D. (2014) 3Dmol.js: molecular visualization with WebGL. Bioinformatics, 31, 1322–1324. 25505090 10.1093/bioinformatics/btu829 PMC4393526

[33] Glass J.I., Lefkowitz E.J., Glass J.S. et al. (2000) The complete sequence of the mucosal pathogen *Ureaplasma urealyticum*. Nature, 407, 757–762. 11048724 10.1038/35037619

[34] Zhuang Y., Yang G.Y., Chen X. et al. (2017) Biosynthesis of plant-derived ginsenoside Rh2 in yeast via repurposing a key promiscuous microbial enzyme. Metab. Eng., 42, 25–32. 28479190 10.1016/j.ymben.2017.04.009

[35] Kelley L.A., Mezulis S., Yates C.M. et al. (2015) The Phyre2 web portal for protein modeling, prediction and analysis. Nat. Protoc., 10, 845–858. 25950237 10.1038/nprot.2015.053 PMC5298202

[36] Chen L., Zhang Y. and Feng Y. (2018) Structural dissection of sterol glycosyltransferase UGT51 from *Saccharomyces cerevisiae* for substrate specificity. J. Struct. Biol., 204, 371–379. 30395931 10.1016/j.jsb.2018.11.001

