# Supplementary material for: GTDB: an integrated resource for glycosyltransferase sequences and annotations
Source: Database (Oxford). 2020 Jun 16;2020:baaa047. doi: 10.1093/database/baaa047 (PMC7296393; doi:10.1093/database/baaa047)
Supplement: supplymentary_file_baaa047 [file supplymentary_file_baaa047.docx]

**Supplementary Materials**


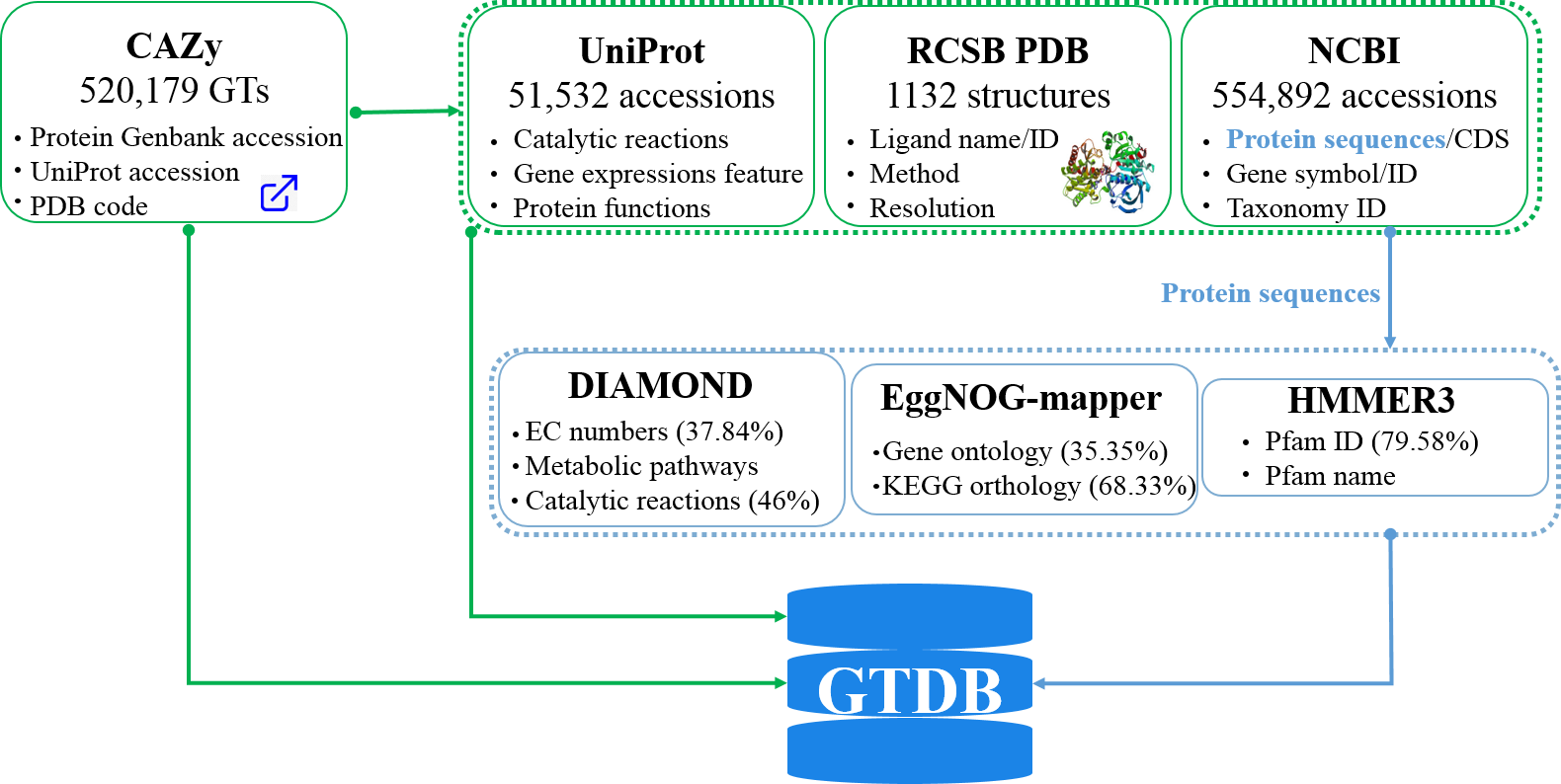


**Figure S1****.** **The workflow of data required in GTDB.**

The contents in green are integrated from third-party database and those in blue are from bioinformatics algorithms.


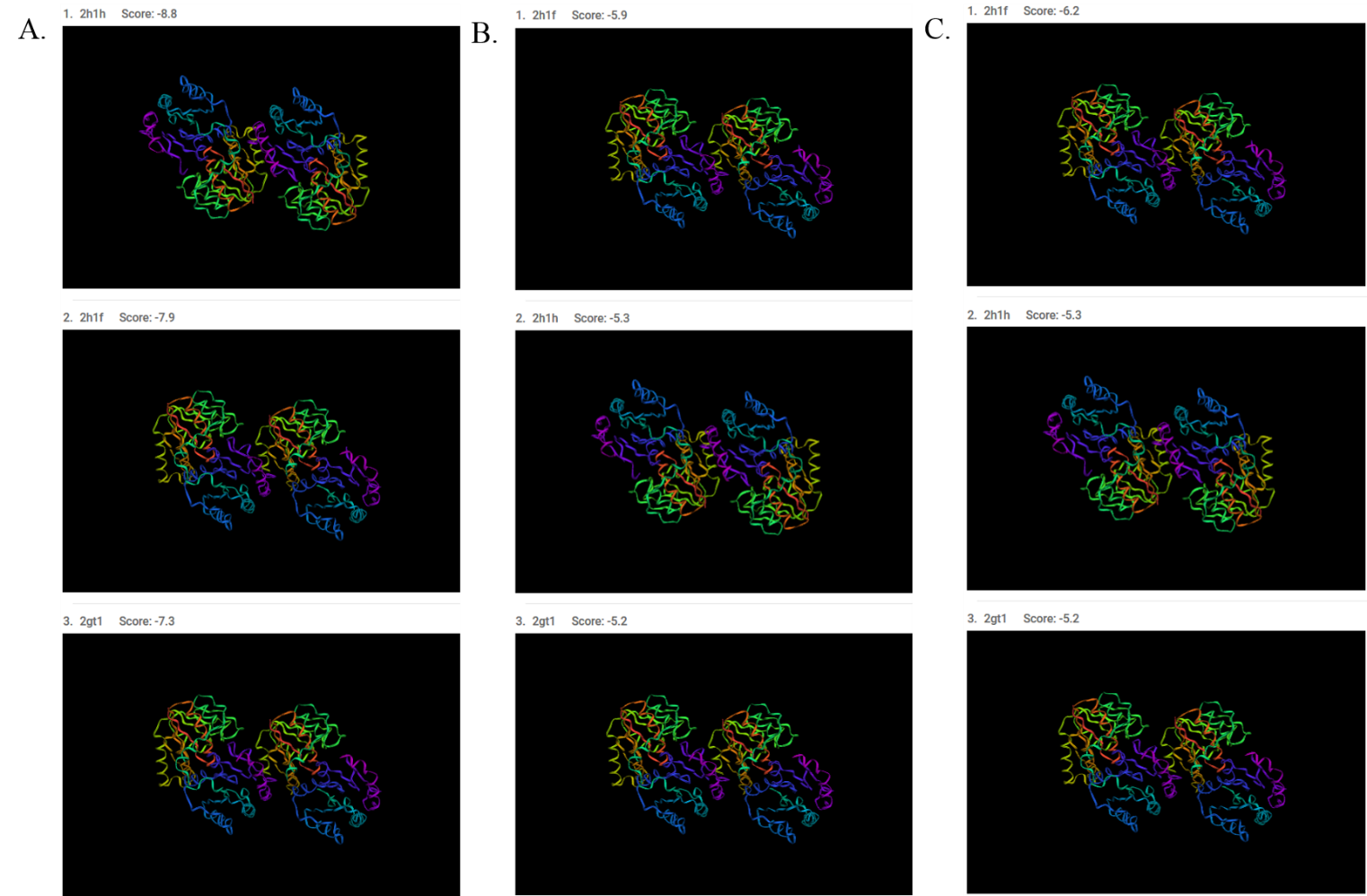


**Figure S2. ADP- L-glycero-D-manno-heptose, D-galactose, D-glucose were docked into entire HepI structure with different configurations (2GT1 and 2H1H are “open” apo HepI structures, 2H1F is the “closed” apo model).**

These docking results are basically consistent with Table S3 in (Bioorg Med Chem Lett. 2018 February 15; 28(4): 594–600. doi: 10.1016/j.bmcl.2018.01.040), which shows that ADP- L-glycero-D-manno-heptose was the tightest binding ligand and shorter-chained alkylated monosaccharides had somewhat weaker binding affinities.

1. The binding modes of ADP- L-glycero-D-manno-heptose and HepI. **B.** The binding modes of D-galactose and HepI. **C.** The binding modes of D-glucose and HepI.


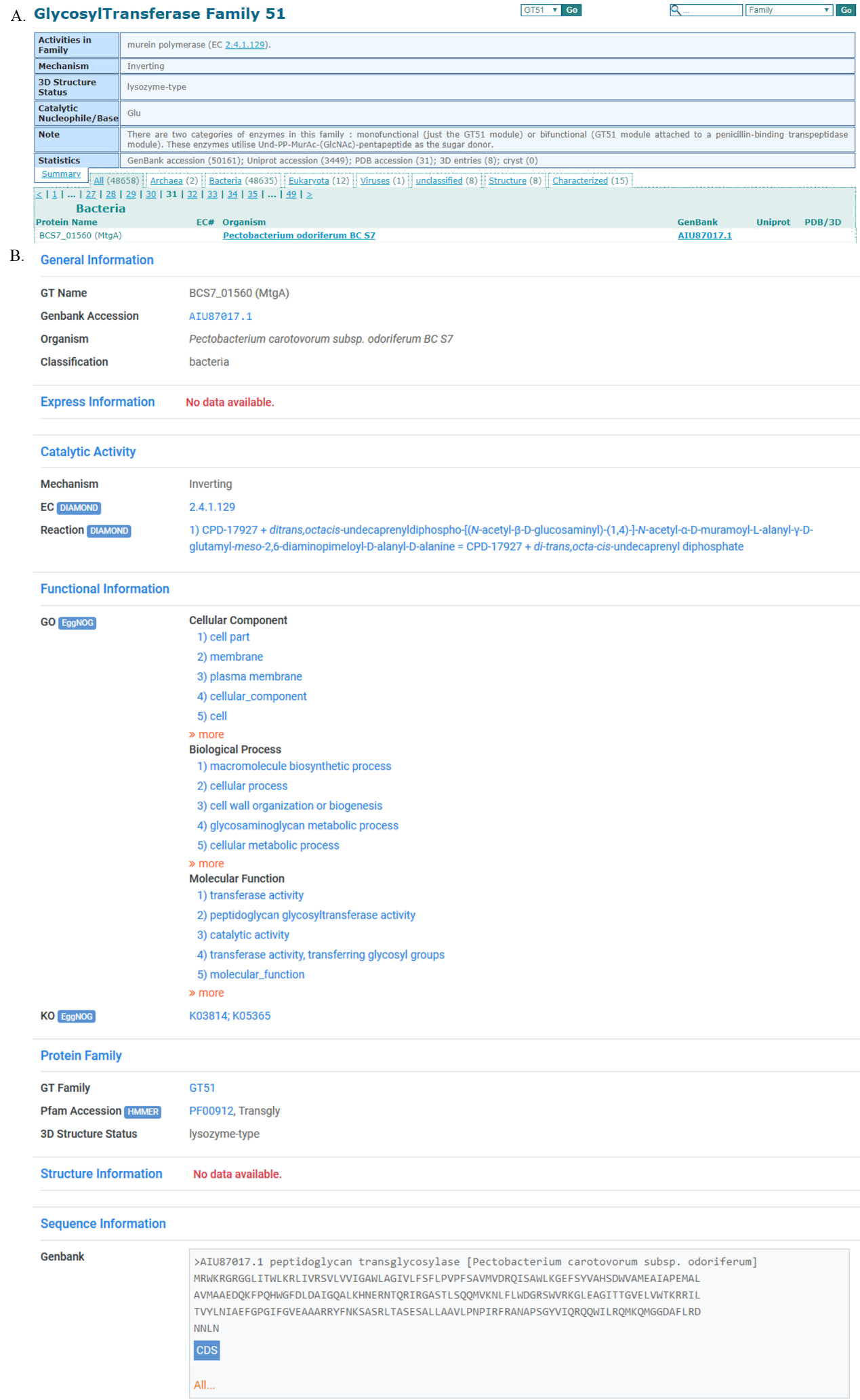
**Figure S3. The information of BCS7_01560 (MtgA) [Pectobacterium carotovorum subsp. odoriferum BC S7] in CAZy database and GTDB, respectively.**

1. The information of BCS7_01560 (MtgA) [Pectobacterium carotovorum subsp. odoriferum BC S7] in CAZy database. **B.** The information of BCS7_01560 (MtgA) [Pectobacterium carotovorum subsp. odoriferum BC S7] in GTDB.
